# Supplementary material for: Characterization of Metal-Specific T-Cells in Inflamed Oral Mucosa in a Novel Murine Model of Chromium-Induced Allergic Contact Dermatitis
Source: Int J Mol Sci. 2023 Feb 1;24(3):2807. doi: 10.3390/ijms24032807 (PMC9917800; doi:10.3390/ijms24032807)
Supplement: Supplementary file 1 [file ijms-24-02807-s001.zip › ijms-2156038-supplementary.pdf]

Supplementary Materials

TRA Ranking Top1-30 of oral mucosa in the ACM Day3

Rank	TRAV	TRAJ	CDR3	%Reads
1	TRAV6-7/DV9	TRAJ32	CALGLYGGSGNKLIF	1.60
2	TRAV11D	TRAJ18	CVVGDRGSALGRLHF(iNKT)	1.31
3	TRAV9-4	TRAJ53	CAVRSGGSNYKLTF	0.87
4	TRAV17	TRAJ52	CALNTGANTGKLTF	0.83
5	TRAV1	TRAJ33	CAVRDSNYQLIW(MAIT)	0.74
6	TRAV13N-4	TRAJ40	CAMEPPGNYKYVF	0.69
7	TRAV10N	TRAJ5	CAASTGTQVVGQLTF	0.61
8	TRAV6D-7	TRAJ12	CALTPGTGGYKVVF	0.59
9	TRAV3D-3	TRAJ35	CAVPTQTGFASALTF	0.56
10	TRAV8-2	TRAJ52	CATDTGANTGKLTF	0.52
11	TRAV10	TRAJ52	CAASTGANTGKLTF	0.50
12	TRAV5-1	TRAJ26	CSASIFNYAQGLTF	0.49
13	TRAV12N-2	TRAJ27	CALSNTNTGKLTF	0.48
14	TRAV4D-4	TRAJ18	CAAGRSALGRLHF	0.47
15	TRAV10	TRAJ39	CAARNNNAGAKLTF	0.47
16	TRAV13-3	TRAJ49	CATGYQNFYF	0.46
17	TRAV7D-2	TRAJ37	CAADPITGNTRKLIF	0.44
18	TRAV7D-2	TRAJ30	CAAKDTNAYKVIF	0.42
19	TRAV6-1	TRAJ11	CVLGDDSGYNKLTF	0.40
20	TRAV6-5	TRAJ33	CALRMDSNYQLIW	0.40
21	TRAV13D-2	TRAJ30	CAIDRITNAYKVIF	0.40
22	TRAV4-3	TRAJ32	CAADYGGSGNKLIF	0.39
23	TRAV4D-3	TRAJ39	CASENRNAGAKLTF	0.39
24	TRAV7-5	TRAJ22	CAMSPASSGSWQLIF	0.38
25	TRAV13-1	TRAJ43	CAINNNNAPRF	0.38
26	TRAV7D-2	TRAJ27	CAASDTNTGKLTF	0.37
27	TRAV8-2	TRAJ26	CATEHNYAQGLTF	0.37
28	TRAV7D-5	TRAJ52	CAVNTGANTGKLTF	0.36
29	TRAV13D-1	TRAJ35	CAMEGFASALTF	0.36
30	TRAV16D/DV11	TRAJ48	CAMREGPDYGNEKITF	0.36

TRA Ranking Top1-30 of oral mucosa in the ACM Day7

Rank	TRAV	TRAJ	CDR3	%Reads
1	TRAV12N-2	TRAJ43	CALSGNNNNAPRF	2.04
2	TRAV12-3	TRAJ26	CALSDFYAQGLTF	1.84
3	TRAV11D	TRAJ18	CVVGDRGSALGRLHF(iNKT)	1.46
4	TRAV13D-2	TRAJ12	CAIGTGGYKVVF	1.38
5	TRAV12N-2	TRAJ18	CALRMDRGSALGRLHF	1.14
6	TRAV9-3	TRAJ34	CAMSNTDKVVF	1.11
7	TRAV9-3	TRAJ42	CAVRLSGGSNAKLTF	1.09
8	TRAV1	TRAJ33	CAVRDSNYQLIW(MAIT)	1.03
9	TRAV7-3	TRAJ30	CAAHDTNAYKVIF	0.98
10	TRAV6-5	TRAJ33	CALSMDSNYQLIW	0.91
11	TRAV4D-3	TRAJ30	CAAAANAYKVIF	0.88
12	TRAV8-2	TRAJ42	CATDPGGSNAKLTF	0.85
13	TRAV9-4	TRAJ34	CAVSPVSNTDKVVF	0.75
14	TRAV9D-4	TRAJ9	CALSARNMGYKLTF	0.72
15	TRAV14D-3/DV8	TRAJ31	CAANNNNRIFF	0.68
16	TRAV4-4/DV10	TRAJ18	CAAEDRGSALGRLHF	0.68
17	TRAV4-2	TRAJ52	CAAANTGANTGKLTF	0.68
18	TRAV14D-3/DV8	TRAJ21	CAASSYNVLYF	0.67
19	TRAV13D-1	TRAJ24	CPTASLGKLQF	0.66
20	TRAV5-1	TRAJ13	CSASANSGTYQRF	0.66
21	TRAV14-1	TRAJ43	CAASGNNNNAPRF	0.63
22	TRAV10N	TRAJ13	CAARNSGTYQRF	0.63
23	TRAV1	TRAJ32	CASYGGSGNKLIF	0.62
24	TRAV12-3	TRAJ22	CALSASSGSWQLIF	0.61
25	TRAV5-4	TRAJ27	CAASATNTGKLTF	0.53
26	TRAV9D-4	TRAJ34	CAMSNTDKVVF	0.53
27	TRAV4D-3	TRAJ24	CAAEAAASLGKLQF	0.53
28	TRAV12N-2	TRAJ13	CALSEHSGTYQRF	0.51
29	TRAV13D-2	TRAJ40	CAIDRTGNYKYVF	0.49
30	TRAV14D-3/DV8	TRAJ30	CAASGSTNAYKVIF	0.49

Figure S1. Top 30 ranking read numbers of TRA clonotypes in the oral mucosa of ACM mice at Days 3 and 7 post-challenge. The distributions of %reads of amino acid sequences of CDR3 regions in the ACM mice showed that the shared iNKT cells (green shading) and MAIT cells (blue shading) in the oral mucosa of ACM mice at Days 3 and 7.

TRB Ranking Top1-30 of oral mucosa in the ACM Day1

Rank	TRBV	TRBJ	CDR3	%Reads
1	TRBV4	TRBJ2-3	CASSRGSAETLYF	0.89
2	TRBV13-3	TRBJ2-2	CASSRDRNTGQLYF	0.62
3	TRBV1	TRBJ1-6	CTCSADSYNSPLYF	0.57
4	TRBV31	TRBJ1-1	CAWTGTPNTEVFF	0.57
5	TRBV29	TRBJ1-4	CASSFDNERLFF	0.55
6	TRBV13-2	TRBJ2-5	CASGDARGDTQYF	0.46
7	TRBV29	TRBJ2-2	CASSFRTGNTGQLYF	0.45
8	TRBV29	TRBJ2-7	CASSWQGVEQYF	0.40
9	TRBV5	TRBJ1-4	CASSQEPGTGPNERLF	0.37
10	TRBV29	TRBJ2-4	CGGGGVGQNTLYF	0.35
11	TRBV16	TRBJ1-2	CASSLGNSDYTF	0.33
12	TRBV31	TRBJ1-5	CAWSPPGQGNQAPLF	0.32
13	TRBV1	TRBJ2-2	CTCSAPGANTGQLYF	0.32
14	TRBV29	TRBJ2-4	CASSLGGRYQNTLYF	0.31
15	TRBV19	TRBJ1-6	CASSIDREYSPLYF	0.30
16	TRBV3	TRBJ2-7	CASSLDGGEQYF	0.30
17	TRBV13-1	TRBJ2-2	CASRGQATGQLYF	0.30
18	TRBV19	TRBJ1-3	CASSAQGNTLYF	0.30
19	TRBV5	TRBJ2-7	CASSQDSRVREQYF	0.29
20	TRBV29	TRBJ1-4	CASSLAGRGNERLFF	0.29
21	TRBV5	TRBJ2-2	CASSQTGTGQLYF	0.29
22	TRBV4	TRBJ1-4	CASSYTGFSNERLFF	0.28
23	TRBV1	TRBJ1-4	CTCSAGQKNERLFF	0.28
24	TRBV13-2	TRBJ2-3	CASGTGASAETLYF	0.28
25	TRBV31	TRBJ2-7	CAWSPGTGGYEQYF	0.28
26	TRBV19	TRBJ2-1	CASSMGTVAEQFF	0.26
27	TRBV15	TRBJ2-1	CASSWGLDWENYAEQ	0.26
28	TRBV19	TRBJ2-7	CASPTGSSYEQYF	0.26
29	TRBV5	TRBJ1-1	CASSQEGTGGTEVFF	0.26
30	TRBV29	TRBJ1-5	CASSFGTDNNQAPLF	0.26

TRB Ranking Top1-30 of oral mucosa in the ACM Day3

Rank	TRBV	TRBJ	CDR3	%Reads
1	TRBV1	TRBJ1-4	CTCSPGQKNERLFF	1.47
2	TRBV5	TRBJ1-4	CASSQAGGTNERLFF	1.19
3	TRBV19	TRBJ1-1	CASSIRTENTEVFF	0.97
4	TRBV31	TRBJ1-6	CAWRTGGYNSPLYF	0.70
5	TRBV14	TRBJ2-3	CASSFEETLYF	0.68
6	TRBV17	TRBJ2-5	CASSRSEEDTQYF	0.57
7	TRBV13-1	TRBJ2-7	CASSEPGQGAREQYF	0.57
8	TRBV1	TRBJ2-5	CTCSARLGEQDTQYF	0.55
9	TRBV13-2	TRBJ1-3	CASGQNSGNTLYF	0.53
10	TRBV19	TRBJ1-1	CASSMEGTEVFF	0.49
11	TRBV13-3	TRBJ2-5	CASSDWGDTQYF	0.46
12	TRBV5	TRBJ1-5	CASSQDGGKRNNQAP	0.45
13	TRBV26	TRBJ1-2	CASSLDNSDYTF	0.44
14	TRBV5	TRBJ2-5	CASSRDWDQDTQYF	0.39
15	TRBV1	TRBJ2-7	CTCSADNYEQYF	0.38
16	TRBV13-2	TRBJ2-7	CASGEPGGAREQYF	0.38
17	TRBV5	TRBJ1-1	CASSQGANTEVFF	0.36
18	TRBV29	TRBJ2-5	CASSSSGLGEDTQYF	0.35
19	TRBV5	TRBJ1-4	CASSQARQGGGERLFF	0.34
20	TRBV13-1	TRBJ1-4	CASSDGRDRGNERLFF	0.34
21	TRBV13-2	TRBJ1-6	CASGDPNRGTPLYF	0.33
22	TRBV14	TRBJ1-6	CASSPPGTNHNSPLYF	0.32
23	TRBV4	TRBJ2-1	CASSYWGGAGAEQFF	0.31
24	TRBV4	TRBJ1-4	CASSFRDRGNERLFF	0.31
25	TRBV13-3	TRBJ1-3	CASSDAWTNSGNTLY	0.30
26	TRBV3	TRBJ2-5	CASSRDWGDTQYF	0.29
27	TRBV13-3	TRBJ2-3	CASSDVAGGAETLYF	0.29
28	TRBV13-3	TRBJ2-4	CASSGGQGTLYF	0.28
29	TRBV4	TRBJ1-3	CASSLGPSGNTLYF	0.28
30	TRBV31	TRBJ2-7	CAWSLVTGAYEQYF	0.28

TRB Ranking Top1-30 of oral mucosa in the ACM Day7

Rank	TRBV	TRBJ	CDR3	%Reads
1	TRBV29	TRBJ2-4	CASTGPNTLYF	1.84
2	TRBV13-2	TRBJ2-1	CASGDKGGAEQFF	0.77
3	TRBV29	TRBJ1-2	CASSLSGQGNSDYTF	0.71
4	TRBV5	TRBJ2-3	CASSPGQGAETLYF	0.70
5	TRBV4	TRBJ2-3	CASSFRDWGPETLYF	0.70
6	TRBV5	TRBJ1-1	CASSHRDRGRTEVFF	0.67
7	TRBV13-3	TRBJ2-1	CASSESGTGGFAEQFF	0.66
8	TRBV5	TRBJ1-1	CASSQGANTEVFF	0.65
9	TRBV5	TRBJ2-3	CASSQEGGRGAETLYF	0.52
10	TRBV5	TRBJ1-1	CASSRQGNTEVFF	0.48
11	TRBV29	TRBJ1-1	CASSPQANTEVFF	0.48
12	TRBV5	TRBJ2-5	CASSQDLNRADQDTQ	0.44
13	TRBV5	TRBJ2-5	CASSPTGGGQDTQYF	0.43
14	TRBV5	TRBJ2-1	CASSQGLGGPYAEQFF	0.43
15	TRBV14	TRBJ1-4	CASSSGQGPERLFF	0.42
16	TRBV13-2	TRBJ1-2	CASGDAQANSDYTF	0.39
17	TRBV20	TRBJ1-4	CGARERQFNERLFF	0.38
18	TRBV19	TRBJ2-4	CASSVTGNTLYF	0.37
19	TRBV5	TRBJ2-5	CASSPQGLGQDTQYF	0.37
20	TRBV29	TRBJ1-3	CASRGTGAGNTLYF	0.36
21	TRBV1	TRBJ2-3	CTCSGQAETLYF	0.35
22	TRBV19	TRBJ2-2	CASSAGRNTGQLYF	0.34
23	TRBV20	TRBJ1-1	CGASWGNTEVFF	0.34
24	TRBV13-3	TRBJ2-3	CASSASGLGGAETLYF	0.34
25	TRBV13-2	TRBJ2-4	CASGEPAGGNTLYF	0.32
26	TRBV29	TRBJ1-4	CASRRQGSFSNERLFF	0.32
27	TRBV14	TRBJ2-1	CASSLRGGYAEQFF	0.32
28	TRBV13-2	TRBJ1-3	CASGDNSGNTLYF	0.32
29	TRBV29	TRBJ2-4	CASSLNTKNTLYF	0.30
30	TRBV13-2	TRBJ1-1	CASGDYNTEVFF	0.30

Figure S2. Top 30 ranking read numbers of TRB clonotypes in the oral mucosa of ACM mice at Days 1, 3, and 7 post-challenge. The Figure showed that the distributions of %reads of amino acid sequences of CDR3 regions of in the ACM mice at Days 3 and 7 post-challenge. There was no shared TRB clone at Days 1,3, and 7 in the oral mucosa of ACM mice.
